# Disease vectors in the era of next generation sequencing

**DOI:** 10.1186/s13059-016-0966-4

**Published:** 2016-05-06

**Authors:** David C. Rinker, R. Jason Pitts, Laurence J. Zwiebel

**Affiliations:** Department of Biological Sciences, Vanderbilt University, Nashville, Tennessee USA; Department of Pharmacology, Vanderbilt Brain Institute, Program in Developmental Biology, and Institutes of Chemical Biology and Global Health, Vanderbilt University Medical Center, Nashville, Tennessee USA

## Abstract

Almost 20 % of all infectious human diseases are vector borne and, together, are responsible for over one million deaths per annum. Over the past decade, the decreasing costs of massively parallel sequencing technologies have facilitated the agnostic interrogation of insect vector genomes, giving medical entomologists access to an ever-expanding volume of high-quality genomic and transcriptomic data. In this review, we highlight how genomics resources have provided new insights into the physiology, behavior, and evolution of human disease vectors within the context of the global health landscape.

## Background

**Fig. 1:**
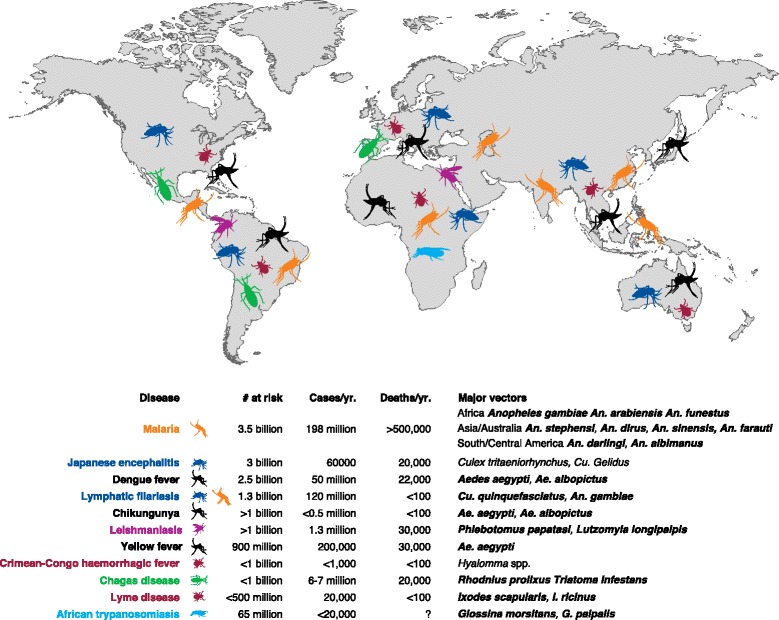
Global distribution of the major vector-borne diseases and associated vectors. Species names in **bold** indicate the current availability of an assembled genome resource

The significance of arthropod vectors in disease transmission came into focus in the late 19^th^ century when researchers such as Charles Alphonse Laveran, Giovanni Grassi, and Ronald Ross convincingly described the role of *Anopheles* mosquitoes in the human malaria cycle. Until that time, it had been largely unappreciated that human diseases could be spread via intermediate organisms (vectors) that could themselves be infected with an agent of human disease. Even 'malaria', the name of the quintessential vector borne disease, is derived from a Latinate word meaning 'bad air' and is reflective of the mystery surrounding the disease’s etiology that persisted for centuries. Subsequent to that initial insight, scores of other arthropod species have been implicated as vectors for many human diseases, and current World Health Organization estimates suggest that 17 % of all infectious human diseases are vector borne (Fig. 1). The broad field of medical entomology emerged on the heels of those early discoveries as scientists sought to examine the biology of insect vectors comprehensively in an effort to reduce their health impact.

Historically, vector control strategies have drawn on biological knowledge about vector species to both curtail their population sizes and limit human contact. Elimination of vector breeding sites was the earliest mechanism of population reduction and this strategy was soon followed by the widespread application of a range of chemical insecticides [1]. Reduction of host–vector contact has also been implemented as a more recent control strategy that uses combinations of chemical (insect repellants) and physical (full-coverage clothing, bed nets, window screens and so on) barriers [2].

More recently, vector control initiatives have also been influenced by the use of increasingly sophisticated computer-modeling approaches, as well as by a rapidly expanding wealth of genetic information and gene-editing technologies. Genetic markers, revealed through molecular karyotyping, polymerase chain reaction (PCR)-based assays and now next generation sequencing (NGS), have led to more detailed systematic and epidemiologic knowledge. Such data, when coupled to advances in computational modeling and more powerful genome-editing technologies, can lead to more accurate estimations of disease risk [3], as well as to the strategic modification of vector genomes to reduce either their competence or their population size [4, 5].

The newest insights into improved vector surveillance and control are increasingly being driven by NGS technologies, which have themselves fostered the rapid accumulation and sharing of genomic resources for 'non-model organisms' such as disease vectors. Indeed, the ever-decreasing cost of NGS has altered the nature and scale of potential scientific queries. It is now very feasible for individual researchers to obtain not just whole-genome sequences for taxa of interest but also genomic information specific to individuals within those taxa. Similarly, comprehensive transcriptional data can be reliably acquired at the level of individual tissue and cell types, while de novo assemblies of transcriptome data can provide a wealth of genetic and phylogenomic information even in the absence of an assembled genome sequence.

In this review, we provide an overview of some of the recent applications of NGS strategies to disease vectors and illustrate how these approaches can inform our understanding of their evolutionary histories, biology, and phenotypes. Studies that have examined a diverse array of vector taxa are discussed. The majority of published research remains focused on mosquitoes (Diptera: *Culicidae*), which by themselves account for the transmission of the majority of the most prevalent vector-borne diseases worldwide (Fig. 1). Nonetheless, NGS technologies have been applied to questions relating to other important vectors, such as triatomine bugs and ticks. More specifically, the studies highlighted here both draw on and impact the abstract concept of vectorial capacity, a quantitative estimation of the degree to which a vector poses a risk to human health. In that light, NGS approaches have been used to examine factors related to vector population sizes (such as reproductive biology and insecticide resistance), the ability of a species to resist infection (vector competence), and the frequency of human–vector contact (host-seeking). We present examples from the published literature covering each of these topics. We conclude by offering some examples of translational research that bring together information garnered from NGS data with ideas for novel vector control strategies. These examples are potential harbingers of the impact that 'big data' will have on the biology of disease transmission.

## Genomics resources for understanding disease vectors

Traditional methods in medical entomology and molecular biology have been the mainstays of vector biology, but new information is needed in the fight against human disease. Genomics technologies offer access to the deeper secrets of organismal biology that are locked in the genetic code, and thus afford tremendous opportunities to increase our understanding of disease vectors. From the basic genomic sequence to nucleotide polymorphisms to profiles of RNA expression, sequencing technologies can be leveraged to probe a wide array of questions about the organization, function, and evolutionary histories of vector genomes. The knowledge gained by having access to entire gene families can inform new vector control strategies in ways that traditional gene-by-gene studies could never hope to. The complete sequencing of the genome of the major African malaria mosquito *Anopheles gambiae* was a milestone effort that ushered in an exciting era in vector biology. More than a decade later, the expansion of both genomic and transcriptomic sequencing capabilities has continued to allow the collection of genomics data from a broad spectrum of vector species.

### Vector genomes

The systematics of arthropods allows for broad distinctions between vector and non-vector species, as well as for inferring those species’ population sizes, ranges, and evolutionary histories. The advent of molecular tools (such as karyotyping, restriction fragment length polymorphism (RFLP) and PCR) has steadily increased the resolving power of species and subspecies identifications down to the molecular level. Of all vector taxa, mosquitoes are the most widely dispersed, with competent vector species present in virtually every geographic cline. Consequently, mosquitoes were early targets for whole genome sequence (WGS) efforts; the 2002 publication of the genome for *An. gambiae* represented a milestone in vector genomics. This paved the way for comprehensive studies of gene function and evolution that have drawn on the complete complement of genes [6]. Early in 2015, the genomes of an additional 16 *Anopheles* species were published [7, 8], an accomplishment that immeasurably benefited from the speed and depth of coverage afforded by NGS. The analysis of the full suite of *Anopheles* genomes, the species makeup of which spans the taxonomic breadth of the entire subfamily, revealed the genomic composition of *Anopheles* mosquitoes to be evolving rapidly. These genomes share tendencies toward X-chromosomal rearrangements distinct from any patterns of gene reshuffling observed in the genomes of the Dengue/Yellow/Zika virus vector mosquito, *Aedes aegypti*, or *Drosophila melanogaster* [8]. Fontaine et al. [9] took advantage of the chromosomal context afforded by the new genomic information to resolve the fine evolutionary relationships that exist within the *An. gambiae* species complex, in which morphological and evolutionary similarity has been difficult to disentangle using conventional methods. By supplementing the new genomic information with additional NGS information derived from individual mosquitoes, a fine-scale evolutionary picture has emerged. This analysis reveals that the two major malaria vector species within the complex (*An. gambiae* s.l. and *An. arabiensis*) were the first to diverge from other minor- or non-vector members of their species complex [9].

Following the release of the *An. gambiae* genome, the genome sequences of *Ae. aegypti* and the West Nile mosquito, *Culex quinquefasciatus*, were published [10, 11]. As was the case for other genome sequencing efforts at that time, the sequencing of these two genomes relied on conventional (Sanger) sequencing technologies and, consequently, were expensive efforts that were time- and labor-intensive. Following the advent of and the steady improvements in NGS technologies, WGS has become an increasingly common undertaking, such that the number of available vector genomes has grown significantly over the past decade. In the past year, the annotated genome sequences of multiple vector and non-vector Anopheline mosquitoes have been made available, resulting in a flurry of ancillary studies [8, 12]. Furthermore, the genome of the Asian tiger mosquito, *Aedes albopictus*, has also recently been completed, offering the potential for timely insights into the genome of this highly invasive Dengue- and Chikungunya-competent vector [13]. Perhaps most telling of the time and effort required to sequence a single insect species’ genome, the genome papers for both *An. stephensi* and *Ae. albopictus* genomes each list just over 30 authors, a number that stands in stark contrast to the 123 authors of the inaugural *An. gambiae* genome report.

Beyond Culicidae, a handful of vector genomes have been assembled and can be accessed at publically available databases, including the National Center for Biotechnology Information (NCBI) and the National Institute of Allergy and Infectious Diseases (NIAID)-supported VectorBase (vectorbase.org). These databases offer an incredible assortment of tools that enable rapid homology searches, sequence downloads, and gene expression analyses. Genomes for the vectors of leishmaniasis, trypanosomiasis, typhus and Lyme disease have been completed, with numerous others in progress. We expect the number of assembled vector genomes to increase rapidly in the next few years as per-genome costs continue to decrease and more studies of neglected tropical diseases are made easier by the relative simplicity of NGS technologies.

### RNA-sequencing and transcriptome assemblies

In addition to the WGS of DNA, massively parallel sequencing of RNA molecules (RNA-seq) has also significantly augmented genome-wide analyses by providing highly quantitative transcript abundance data, as well as a wealth of sequence, isoform, and expression information for the vast majority of encoded genes in a vector species [14]. Importantly, because RNA-seq largely captures only fully spliced transcripts, an informative de novo transcriptome assembly of RNA sequences can be generated affordably and analyzed efficiently, even in the absence of an assembled genome. Already, de novo assemblies of RNA-seq-derived insect transcriptomes have provided invaluable sequence information amenable to powerful molecular evolutionary analyses and quantitative gene expression profiles in the absence of genome resources (reviewed in [15]). For vector insects, the recently completed housefly (*Musca domestica* L.) [16] and tsetse fly (*Glossina morsitans*) [17] genome projects both used transcriptome assemblies as a means of enriching the quality of their respective genome assemblies, at least insofar as transcribed regions are concerned. Additionally, in mosquitoes, de novo transcriptome assemblies were successfully applied to *An. funestus* well in advance of the availability of a genome sequence [18].

In *Culicinae*, the transcriptome assembly of the non-bloodfeeding genus *Toxorhynchites* has revealed extensive evidence for its phylogenetic relationship relative to the two fully sequenced major *Culicinae* vectors, *Ae. aegypti* and *Culex pipiens* [19]. In ticks, only the Lyme disease vector, *Ixodes scapularis* [20], has an assembled genome, but robust transcriptomic approaches have been used for the study of other important vector species, such as *Ixodes ricinus* [21, 22], *Amblyomma americanum* [23], *Dermacentor variabilis* [24, 25], and *Hyalomma marginatum rufipes* [26] (reviewed in [27]). Comparative studies of vector transcriptomes can be expected to provide important insights into the shared features of common biological processes, as well as the identification of species-specific transcripts that may ultimately be targeted for the design and development of novel control strategies. Notable advantages of de novo transcriptome assembly are its rapid turnaround time and relatively low acquisition cost. For example, at present, the only genome assembly for a vector of Chagas disease is that of the kissing bug, *Rhodnius prolixus*, a species that has already been eradicated in some Chagas-endemic regions of Central America. Nonetheless, *Triatominae* vectors other than *R. prolixus* are emerging as potentially new Chagas vectors, and a composite strategy toward de novo transcriptome assembly has proved effective in capturing some of the transcribed genomic elements of *Triatome brasiliensis*, an emergent Chagas vector in Brazil [28].

## Improved understanding of vector biology

**Fig. 2:**
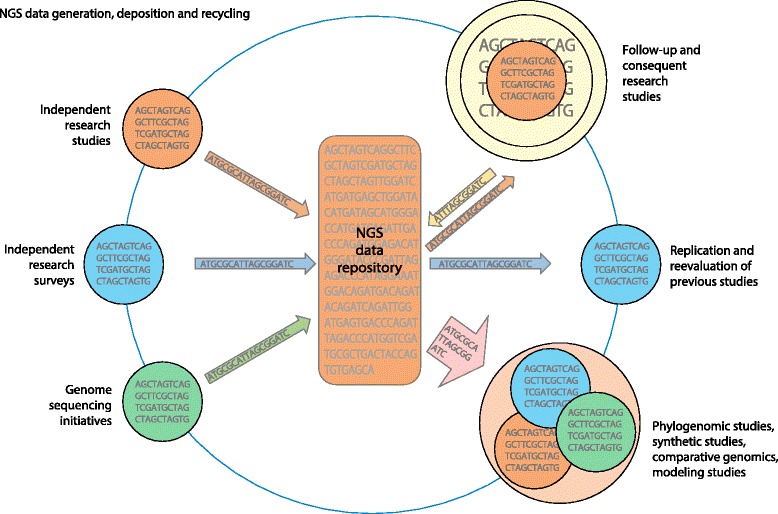
Data sharing potential of next generation sequencing (*NGS*) information. Independent research initiatives generate large volumes of NGS data that are deposited in public archives. Once deposited along with their metadata, these raw reads may go on to serve any number of future roles that supplement and facilitate subsequent research efforts by independent groups. If properly curated and annotated, these NGS data may be reused in any number of productive ways resulting in an overall enrichment of genomic information from which future research studies can benefit

The increasing availability of NGS- and WGS-derived metadata represents a watershed opportunity to transform research efforts that touch upon many, if not all, salient aspects of vector biology. One can envision a landscape in which publically available datasets are continuously augmented by a wide range of sources encompassing small-scale, single-species independent research, moderate-scale multi-species surveys, and large-scale network-level initiatives. In turn, these new datasets may be shared, supplemented and integrated with one another, facilitating the efficient follow-up of previous studies and fostering previously unforeseen efforts for synthetic studies (Fig. 2). As a result, data sharing of NGS information holds the potential to spur the development of novel approaches to reduce both vector competence and vectorial capacity across a wide spectrum of disease vector insects.

### Reproduction and vector populations

NGS and WGS datasets have informed studies involving the reproductive biology and developmental trajectories of disease vectors, highlighting potential vector population control strategies. A particularly salient example is the recent identification and characterization of *Nix* as a male-determining factor in *Ae. aegypti*, which was facilitated by sequencing and comparing male and female genomes to identify male-specific genomic regions. An RNA-seq-based filter applied to these regions then highlighted the *Nix* gene, a distant homolog of *D. melanogaster tranformer-2*, which is known to play an important role in that species’ sex determination. Subsequent knockout and ectopic expression studies confirmed the role of *Nix* as a male-determining factor in *Ae. aegypti* [29].

While the reproductive biology of *Ae. aegypti*, *An. gambiae,* and *G. morsitans* share similarities such as single, conspecific matings (monandry) and nutritive triggers (blood consumption) that stimulate egg development (reviewed in [30]), reproduction in *G. morsitans* is distinguished by intrauterine larval development and maternal nourishment, termed adenotrophic vivipary. As a potential consequence of this novel reproductive strategy, the *Glossina* genome [17] displays an expansion in the number of milk-protein genes that mirrors the expansion of similar genes in mammals. This finding highlights the genomic underpinnings of lactation physiology and provides potential targets for tsetse-specific control [31]. In the American dog tick *D. variabilis*, a recent transcriptomic-based study of the testes, vas deferens, and accessory glands of adult males has identified numerous transcripts for genes that are likely to be involved in spermatogenesis and other reproductive functions. These findings were extended using proteomics to identify a set of peptides that strongly correlated with expressed transcripts related to reproduction [25], an area of intensive study in insect vectors. The future application of studies such as these could be the development of compounds that disrupt fertilization or perhaps mating in vector species. For example, one can envision the application of spermatogenesis-disrupting compounds proximate to or directly onto host species as a means of reducing tick populations.

The ability of a vector population to endure seasonal climatic shifts is an attribute that directly impacts the ability of vectors to transmit diseases over broad ranges. One of the most important mechanisms for overwintering in mosquitoes is a shift toward developmental dormancy, called diapause, which allows the organism to maintain a breeding population during periods when temperatures and host availability are unfavorable. The development stage of diapause varies among species, and the molecular basis of the shift toward diapause is imperfectly characterized (reviewed in [32]). Nevertheless, photoperiodic diapause is an important predictor of how readily invasive species can spread. For example, egg diapause is prominent in temperate populations of *Ae. albopictus* [33]. The mechanisms of photoperiodic diapause in *Ae. albopictus* have been successfully explored by sequencing the whole transcriptome of developing oocytes and assembling genes de novo [34, 35]. The results of this transcriptome-based analysis ultimately pointed to preparatory signatures of photoperiodic diapause that are unique to *Ae. albopictus*, suggesting that a plausible, reproductive adaptation has facilitated the global proliferation of this vector. Importantly, this work was achieved prior to the release of the *Ae. albopictus* genome assembly.

Beyond basic genomics and transcription studies, population-level variation within and between vector species can also be characterized by applying the power of NGS. In *C. pipiens*, RNA-seq proved to be an expedient tool for highlighting genetic components involved in local adaptation by measuring the divergence rates of genes between the morphologically identical wild (*C. pipiens*) and domesticated (*Culex molestus*) forms [36]. Similar approaches in *An. gambiae* s.l. and *Ae. aegypti* have also proved fruitful [37, 38]. In an elegant application of sequencing technology to vector biology, Quillery et al. [39] utilized a genome-reduction strategy and pyro-sequencing to generate short-read libraries from two populations of *I. ricinus*. A large number of single nucleotide polymorphisms (SNPs) were identified, a subset of which were sampled for variation revealing deviations from Hardy-Weinberg equilibrium among six field-collected populations. Restriction-site associated DNA marker (RAD) sequencing is another genome-reduction strategy that can provide efficient population genomic data for non-model species. RAD sequencing of *Ae. aegypti* specimens from around the world suggests that a single subspeciation event occurred within the domesticated form in Africa; the mosquitoes then dispersed globally along commercial trade routes [40]. Both studies validate the likelihood that these methodologies will be useful for assessing population genetic structures in non-model vector species, with or without genome assemblies. Finally, it is important to note that the Wellcome Trust (via its Sanger Institute) has established the *Anopheles gambiae* 1000 genomes (Ag1000G) consortium (https://www.malariagen.net/projects/vector/ag1000g) to provide a global repository for WGS data collected from wild-caught mosquitoes across Africa, thereby providing a catalog of genetic variation across natural vector populations. Given its scope and the otherwise nearly impossible access to such datasets, Ag1000G is almost certain to become an increasingly important resource for the analysis of vector competence and vectorial capacity in *An. gamb*iae.

## The genomics of host-seeking behavior

The concept of vectorial capacity considers the totality of vector–host–parasite interactions, including the proclivity of a vector species to feed preferentially on a given host, the vector population size, susceptibility to infection, and survival rates [41]. The host-preference parameter, specifically the degree of anthropophagy (human biting), will dramatically impact the rate at which host-specific pathogens spread. Conversely, vectors that blood feed more opportunistically will transmit any host-specific pathogens less efficiently.

Long- and medium-range host-seeking behaviors, which are loosely defined as occurring outside a radius of a few meters around hosts, are directly attributable to host-associated olfactory and other chemosensory cues and dramatically impact the vectorial capacity of insects. Accordingly, the molecular underpinning of chemosensation has been a major area of focus for both basic study and vector control. Because components of the chemosensory signal transduction machinery are generally highly localized within specific appendages, such as the antennae, maxillary palps, and labellum, tissue-specific transcriptome profiles have become essential for measuring transcript abundances that would otherwise be diluted and potentially undetectable in whole-body preparations of RNA. In contrast to the relative paucity of studies on gustatory appendages, chemoreceptor abundance quantitation by RNA-seq has been carried out comprehensively in many of the major olfactory appendages of *An. gambiae* [42]*, C. quinquefasciatus* [43]*, Ae. aegypti* [38], *An. quadriannulatus* [37], *Cimex lectularius* [44], and *T. brasiliensis* [28].

An initial RNA-seq study of *An. gambiae* antennae showed that levels of chemoreceptors were lower overall in males than in females. This observation is largely accounted for by the antennal sexual dimorphism that exists between blood-feeding females and the non-blood-feeding males, but the RNA expression data also revealed the surprising finding that the variety and relative abundances of the chemoreceptors were otherwise remarkably conserved between the sexes [42]. Also in *An. gambiae*, an RNA-seq time-course study revealed the relationship between chemoreceptor transcriptional differences and reproductive biology. Rinker et al. [45] observed that individual odorant receptor (OR) transcript profiles in female antennae changed only slightly on the taking of a blood meal but, when viewed collectively, the cumulative effects of these small changes showed that there was an overall shift in olfactory responsiveness in advance of ovipositing. Similarly, subtle distinctions in OR transcriptome profiles were reported in two studies that compared mosquito species that were phenotypically divergent in terms of their host preferences. The first comparison between *An. gambiae* and its less-anthropophilic sibling species *An. quadriannulatus* pointed to an overall enrichment of transcripts for multiple ORs in *An. gambiae* that are predicted to act in concert to enhance responsiveness to human-associated odors [37]. In the second study, differences in the antennal transcriptome profiles of two subspecies of *Ae. aegypti* that differ in their preferences for feeding on humans were examined. The results pointed to notable differences in both the abundance and functional response of a single OR, suggesting that it might be linked to the anthropophily exhibited by one subspecies [19, 38]. Whether or not the different degrees of anthropophily in *Ae. aegypti* can be attributed to only a single chemoreceptor rather than to a suite of chemoreceptor genes or other genes remains an open question.

In contrast to those of mosquitoes, the antennae of the bedbug *C. lectularius* have very few olfactory sensing hairs (sensilla) and accordingly also appear to express relatively few ORs [44]. This finding is commensurate with the ectoparasitic lifestyle of bedbugs in that they spend their lives in immediate proximity to their hosts and thus do not engage in long-range host seeking. The larger picture that emerges from these whole-transcriptome studies is that the chemoreceptors transcribed in sensory appendages in both vector and non-vector insects reflect the olfactory requirements of the organism. Alterations in the levels of these transcripts that afford a degree of chemoreceptive plasticity could further modulate peripheral olfactory signals in response to physiologic and biotic cues. Such a mechanism could also be an expedient for niche adaptation that is integral to speciation. Importantly, because alterations in the transcriptome profile typically occur without any change within the organism’s underlying chemosensory gene repertoire, quantitative RNA-seq-based studies of chemosensory tissues represent an essential tool for the examination of subtle, yet profoundly important, olfactory-related phenotypic differences.

## Genomics of immunity

Vector arthropods and the diseases that they transmit are the evolutionary byproducts of complex host–parasite, genotype-by-genotype interactions. Successful vector-borne parasites are finely tuned to negotiate the physiologies and immune responses of not just one but two hosts, and comprise one corner of a complex 'co-evolutionary triangle'. In the case of malaria, the genomes of the *Plasmodium* pathogen, the *Anopheles* vectors, and human hosts all bear testament to this three-way tug-of-war [46, 47]. Given that the degree of vector competence within Anophelinae can be highly heterogeneous, even among closely related sister subspecies, the genomic factors impacting the degree of competency are likely a myriad. In this light, it is credible that whole-genome approaches represent a unique opportunity to acquire new insight into this multifaceted interaction.

For most vector species, only a minority of individuals are infective at any given time. This is partially due to heterogeneity in vector immune responses, although the evolutionary origins of such heterogeneity are not always clear. In *An. gambiae*, adult susceptibility to *Plasmodium* infection may have complex origins in larval adaptations to challenges stemming from microbial diversity among breeding sites [48]. Although loci associated with defense against *Plasmodium* appear to be under strong selective pressures [49] and *Plasmodium* effectively suppresses specific aspects of the mosquito’s defense mechanisms [50], divergences in the selective signatures of other anti-parasitic genes suggest that *Plasmodium* was not the principal evolutionary driver [51, 52]. Recently, a fascinating co-evolutionary link between reproduction and immunity was found in *Anopheles* mosquitoes using new genomic data [53]. This comparative genomic study found that a male-derived steroid hormone and its female-derived interacting protein appeared to have evolved in concert, and that, upon mating, the male hormone induced an increase in lipid transporters that are known to inhibit the anti-*Plasmodium* immune response in females. Beyond such specifics, recent RNA-seq studies in *An. gambiae* have indicated that much of the known transcriptional response to parasite infection stems from unannotated genomic regions, leading to speculation that the mosquito may be employing non-coding RNAs as part of its basic response mechanism [54, 55].

Furthermore, WGS/NGS-based approaches have increased the awareness of the role played by the native microbiome of vector insects in the modulation of the immune response. Most notably, *Wolbachia* infection of several mosquito species has been implicated in the dramatic reduction of vector competence for *Plasmodium* pathogens and for a number of arboviruses (including those responsible for Dengue, Yellow, Chikungunya, West Nile, and Zika fevers) [56–59]. In *An. gambiae*, the mosquito's tolerance for harboring midgut bacteria can aid in abating the immune response to *Plasmodium* [60], although some bacteria have been shown to confer resistance by directly interfering with the development of the parasite [61]. In *Ae. aegypti*, a three-way interaction was observed among microbiome composition, mosquito immune response, and Dengue virus infection [62], echoing tripartite interaction similar to that reported for trypanosome infection in *G. morsitans* [63]. In *R. prolixus*, high-throughput pyrosequencing allowed an accurate time-course quantitation of gut microbiota, revealing that the presence of *Trypanosoma rangeli* pathogens directly affects microbiome composition, probably by interacting with and affecting the responsiveness of the bug’s immune system [64]. Given the complex immunomodulatory role that gut microbiota appear to play in vector competency, WGS/NGS-based approaches offer tremendous potential for future work to further elucidate these multi-organism interactions.

The blacklegged tick, *I. scapularis*, transmits numerous human pathogens, including the causative agent of Lyme disease in North America, *Borrelia burgdorferi*. Although understudied, immunity genes are likely to contribute to gut microbe homeostasis in ticks, and thus influence the competence of these vectors for human pathogens. A recent review identified a set of 234 immunity-related genes in the *I. scapularis* genome using bioinformatics searches with queries derived from the genome annotation and published literature [65]. While observational in scope, studies such as these lay an important foundation for future work on comparative phylogenomics and the effects of immune responses on pathogen survival. A recent deep-sequencing-based study has characterized the transcriptome of *I. ricinus* hemocytes derived from partially engorged females [21]. This study identified more than 300 transcripts that were significantly overrepresented in these cells, including transcripts encoding antimicrobial peptides and pathogen-recognition proteins [21]. These representative impact areas illustrate how transformative the advent of NGS/WGS approaches can be within diverse areas of vector biology. Such advances give rise to additional challenges in incorporating these resources into meaningful areas of study, and in identifying new targets that can be harnessed in the development of novel control strategies.

## The future of vector genomics and vector control

Translational studies that bridge the gap between genomic data and real-world applications are both present and forthcoming. Several recent innovations demonstrate how genomic data can foster the design of new vector-management tools. In one example, a chemical compound was identified that activates the highly conserved insect odorant receptor co-receptor (Orco) and, thus, has the potential to interfere with the host-seeking behaviors of insect vectors by hyper-stimulating olfactory sensory neurons [66]. In a second example, a small molecule inhibitor of a human inward rectifier potassium channel (Kir) was found to block the activity of a mosquito Kir; this molecule might provide the structural basis for novel insecticide design [67]. Finally, in a more publicized example, genetically modified male mosquitoes are being actively deployed to promising effect in suppressing natural vector populations [68]. Because the development of these control strategies has been directly facilitated by the ready availability of genomic resources, it is reasonable to posit that increasing genomic resources will also further inform future control strategies. This effort will continue to identify new targets [69] for chemical control or will facilitate the genetic engineering of incompetent vectors through the selective alteration of genes that are implicated in vector competency (for examples, see [70–73]).

Moreover, NGS technologies will be increasingly harnessed for population-level monitoring of nascent and emerging vectors. As has already been witnessed in the spread of insecticide resistance or in the elimination of *R. prolixus* from Central America (reviewed in [74]), new genetic variants will invariably emerge to perpetuate the risk to human health. Effective monitoring of vector populations using NGS strategies should allow for more rapid identification of emerging trends and for the development of better predictive models to forecast these trends within vector populations [75]. As sequencing technologies and data-analysis approaches continue to become faster and more accessible, assays of pooled samples of individuals [76–78] will facilitate the comprehensive monitoring and mapping of vector species. The resulting datasets will incidentally contain valuable genetic information relating to infection rates and potentially (via sampling of blood meals) host preferences. Importantly, these data retain their value indefinitely as they can be analyzed retroactively. For example, novel functional variants can be identified to help trace the origins of emergent insecticide resistance. Finally, such multidimensional datasets, which could conceivably be collected and sequenced with relative ease and economy over large spatiotemporal ranges, could then better inform models of disease transmission and risk that may be lacking in contextual sophistication [75]. Initiatives aimed at curbing vector populations through habitat manipulation or the application of insecticides have met with some success, but these strategies also provide selective pressure that can result in genomic adaptations that severely impair future control efforts. This is most dramatically illustrated by the global rise of insecticide-resistant insects [79]. The knockdown resistance mechanism, which results from mutations in the molecular targets of ubiquitously over-applied pyrethroid insecticides, has arisen independently in most arthropods and is thus readily detectable using conventional PCR-based strategies [80]. However, insects can also become resistant to insecticides through other cryptic metabolic mechanisms that break down the insecticidal agents and render them inert, especially those involving members of the cytochrome p450 family of monooxygenase enzymes [81]. The mechanistic changes leading to this mode of resistance are less understood and believed to be much more species specific [82–84], making nascent resistance difficult to detect across diverse populations (for example [85]). NGS can provide routes toward agnostic implication of how metabolic pathways have evolved to confer insecticide resistance. For example, a recent RNA-seq study in insecticide-resistant *Ae. aegypti* has uncovered numerous genomic changes (including polymorphism, copy number variation, and gene amplification events) in certain detoxification enzymes that could then serve as informative markers for monitoring emerging resistance through simple PCR-based assays [86].

## Conclusions

The past several years have witnessed the increasingly rapid adoption of NGS technologies to address questions relevant to the biology and evolution of disease vectors. WGS efforts have resulted in full genome sequences for most of the major arthropod vector species. For more neglected species, de novo transcriptome assembly from RNA-seq data has been sufficient to reveal coding sequences, SNPs, and differential expression. As these data continue to be generated, they should be made available to other researchers through public databases such as NCBI’s Sequence Read Archive (SRA), the European Nucleotide Archive (ENA) and the DNA Data Bank of Japan (DDBJ). In this way, the work of one research group not only informs the study at hand but can also be mined to address innumerable future questions (Fig. 2). Furthermore, the field of vector biology would be well served by the adoption of a set of common data standards that could provide a basic framework to ensure that high-quality, readily accessible datasets will be optimized in their utility to other researchers. This could be accomplished by first examining the standards that groups such as the Immunogenomic Next Generation Sequencing Data Consortium (http://igdawg.org/ngs.html) have put forward. In this way, the true power of large repositories of NGS data can be fully utilized so that the data are both particularly and cumulatively informative, becoming a gift that keeps on giving.

We have attempted to highlight the growing impact of NGS on vector biology. Nonetheless, it is clear that too few studies have utilized sequencing-based approaches despite their rapidly expanding accessibility. It will probably be some time before the field of medical entomology embraces the comprehensiveness and agnosticism offered by NGS assays. Until that happens, the potential benefits of data integration among studies will remain unrealized, and the myriad potential of this 21^st^ century research strategy will remain mired within the experimental paradigms of the 20^th^ century.

## Abbreviations

Ag1000G: *Anopheles gambiae* 1000 genomes consortium
Kir: human inward rectifier potassium channel
NCBI: National Center for Biotechnology Information
NGS: next generation sequencing
OR: odorant receptor
PCR: polymerase chain reaction
RAD: restriction-site associated DNA marker
RNA-seq: massively parallel sequencing of RNA molecules
SNP: single nucleotide polymorphism
WGS: whole genome sequence
